# Blastic plasmacytoid dendritic cell neoplasm of the breast: A case report and review of the literature

**DOI:** 10.1097/MD.0000000000025699

**Published:** 2021-05-14

**Authors:** Hyo-jae Lee, Hye Mi Park, So Yeon Ki, Yoo-Duk Choi, Sook Jung Yun, Hyo Soon Lim

**Affiliations:** a Department of Radiology; b Department of Pathology; c Department of Dermatology, Chonnam National University Hwasun Hospital, Chonnam National University Medical School, Hwasun-gun, Jeollanam-do, Republic of Korea.

**Keywords:** blastic plasmacytoid dendritic cell neoplasm, breast, mammogram, ultrasound

## Abstract

**Rationale:**

Blastic plasmacytoid dendritic cell neoplasm (BPDCN) is an uncommon and aggressive hematologic malignancy that arises from plasmacytoid dendritic cells. BPDCN typically presents with skin lesions with or without involvement of lymph nodes, peripheral blood, or bone marrow. However, breast involvement of BPDCN is rare and there has been no report describing the radiologic features of BPDCN within breast parenchyma.

**Patient concerns:**

We report a case of a 47-year-old woman who presented with an incidentally detected hypermetabolic breast lesion on PET/CT with concurrent right cheek plaque.

**Diagnoses:**

Skin biopsy was performed for the right cheek plaque. Mammography and breast ultrasonography were performed to evaluate the breast lesion. The lesion was depicted as a 2.5 cm sized focal asymmetry on mammogram and an irregular heterogeneous echoic mass within the mammary zone of the right upper outer breast. Core needle biopsy was performed for the breast lesion. Histologic diagnosis of the two lesions was BPDCN.

**Interventions:**

The patient was treated with induction and consolidation chemotherapy and received allogenic peripheral blood stem cell transplantation.

**Outcomes:**

The patient remains in complete remission state without relapse at 34 months since initial diagnosis.

**Lessons:**

This is the first case of BPDCN manifested as a breast parenchymal mass and assessed by diagnostic breast imaging tools (mammography and ultrasonography). This case report is significant for BPDCN within the breast parenchyma and presenting rare radiologic description of BPDCN.

## 1 Introduction

Blastic plasmacytoid dendritic cell neoplasm (BPDCN) is a rare disease that accounts for approximately 0.44% of primary hematologic neoplasm and 0.7% of primary cutaneous lymphomas.^[1,2]^ BPDCN has a high predilection for skin involvement; it commonly presents as cutaneous and subcutaneous nodules, erythematous macules, or papules.^[3]^ Other atypical manifestations including liver, lung, paranasal sinus, tonsil, testis, and central nervous system involvement have also been described in the previous literatures.^[4,5]^ Meanwhile, few cases of BPDCN involving the breast have been reported (Table 1); Borchiellini et al^[6]^ reported the first case of BPDCN as a breast nodule, and Chen et al^[7]^ reported a case of a soft tissue mass in the chest wall that was confirmed as BPDCN. However, to the best of our knowledge, there has been no report describing the radiologic features of BPDCN within breast parenchyma. Herein, we report rare imaging characteristics of BPDCN that presented as a breast parenchymal lesion in a 47-year-old woman concurrently diagnosed with BPDCN of the right cheek. A review of the previous literatures and the clinicopathologic features and treatment of BPDCN are also presented.

**Table 1:** Summary of published cases of BPDCN with breast involvement.

Case No.	Reference	Age	Sex	Symptoms	Therapy	Outcome
1	Borchiellini et al ^[6]^	60	F	Subcutaneous nodule	CTx, allogenic BMT	ACT
2	Chen et al ^[7]^	39	F	Mass with ulceration	CTx	Transfer
3	Kim et al ^[15]^	40	F	Palpable mass	RTx, CTx	ACT
4	Jeong et al ^[16]^	53	M	Growing skin lesion	Palliative RTx	Transfer
5	Kim et al ^[17]^	40	F	Palpable mass	CTx, RTx	ACT
6	Safaei et al ^[18]^	61	M	Palpable mass	CTx	ACT

ACT = alive in complete remission, BMT = bone marrow transplantation, BPDCN = blastic plasmacytoid dendritic cell neoplasm, CTx = chemotherapy, RTx = radiation therapy.

## 2 Case report

This case was approved by the Institutional Review Board (IRB) of Chonnam National University Hwasun Hospital (IRB No. CNUHH-2020–139) and informed consent was waived.

**Figure 1:**
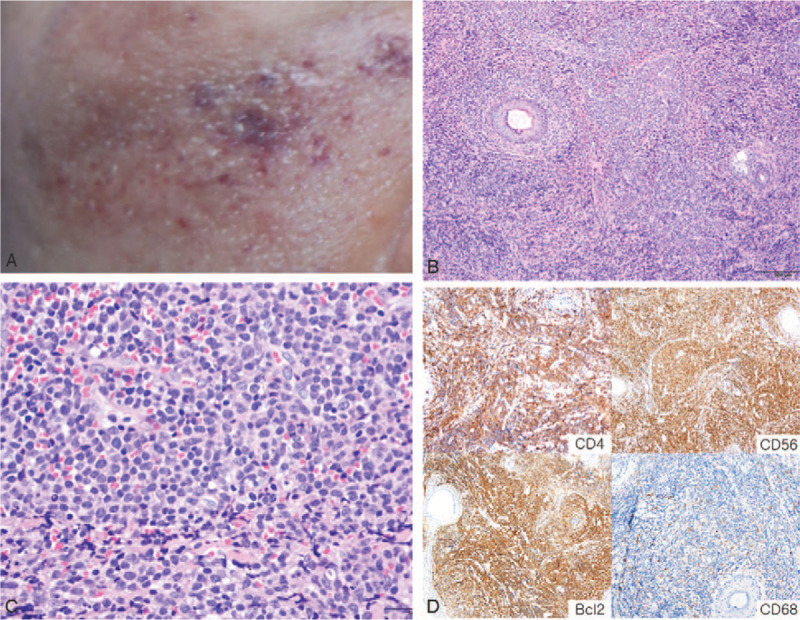
Blastic plasmacytoid dendritic cell neoplasm of the right cheek. (A) A 4.5 × 4.2-cm-sized brownish to violaceous plaque is observed in the right cheek. (B) Microscopically, the lesion shows a diffuse monotonous infiltrate of immature lymphoid cells in the dermal layer (H&E stain, × 20). (C) The cells have an irregular nuclear contour, fine chromatin, small nucleoli, and scanty cytoplasm (H&E stain, × 200). (D) The cells have immunoreactivity for CD4, CD56, Bcl2, and focal immunoreactivity for CD68. (×100 for CD4 and CD68; × 40 for CD56 and Bcl2).

A 47-year-old woman presented with a 6-month history of a 4.5 × 4.2 cm-sized brownish to violaceous right cheek plaque (Fig. 1A). She had no specific medical or psycho-social history or family history of genetic disorders. The lesion was not painful or tender, but the surrounding skin had hardened. She did not receive any therapeutic interventions before with her cutaneous findings. She underwent skin biopsy of the right cheek lesion. Microscopically, the lesion revealed dermal diffuse infiltration of immature lymphoid cells (Fig. 1B). The tumor cells had irregular nuclei, fine chromatin, and scanty cytoplasm (Fig. 1C). Necrosis or vascular invasion were not typical. On immunohistochemical (IHC) staining, the tumor cells were positive for CD4, CD56, leukocyte common antigen (LCA), and Bcl2, focally positive for CD68, terminal deoxynucleotidyl transferase (TdT), and negative for any specific markers of common myeloid, T-cell, B-cell, or monocytic-cell lineages (CD3, CD10, CD20, CD34, MPO) or Epstein-Barr virus encoded RNA (Fig. 1D). The skin lesion was thus diagnosed as BPDCN. She was transferred to hematology department of our hospital for further evaluation and treatment.

**Figure 2:**
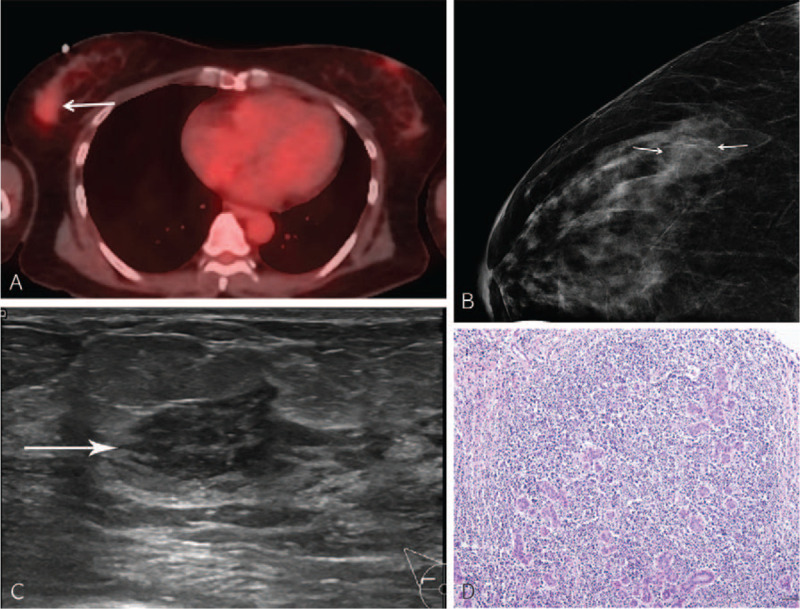
Blastic plasmacytoid dendritic cell neoplasm of the right breast. (A) ^18^F-FDG PET-CT shows a mild hypermetabolic lesion (_max_SUV = 1.9; arrow) in the upper outer quadrant of the right breast. (B) Mammogram shows a 2.5-cm-sized focal asymmetry (arrows) in the right outer breast. (C) Ultrasonogram shows a 3.1-cm-sized irregular heterogeneous echoic mass (arrow) in the mammary zone of the right breast, which corresponds to the lesion observed on PET-CT. (D) Microscopic examination shows diffuse infiltrates of small immature lymphoid cells, which is pathologically identical to the specimen from the right cheek and it is diagnosed as blastic plasmacytoid dendritic cell neoplasm (H&E stain, × 100).

Complete blood cell count results revealed lymphocytosis (57.2% (normal range, 20–40%)) and mild monocytosis (12.2% (normal range, 2–10%)). Bone marrow biopsy and aspiration smear did not show definitive immature cells in the specimen. There was no chromosomal abnormality. The patient underwent fluorine-18-fluorodeoxyglucose positron emission tomography-computed tomography (^18^F-FDG PET-CT) for staging. PET-CT revealed a mild FDG-avid lesion (_max_SUV = 2.2) on the right cheek. Incidentally, a 3-cm-sized focal hypermetabolic mass lesion (_max_SUV = 1.9) was detected in the right breast (Fig. 2A). Mammography and breast ultrasonography were performed to evaluate the mass. The mammogram revealed a 2.5-cm-sized focal asymmetry in the upper outer quadrant of the right breast (Fig. 2B). Moreover, ultrasonogram revealed a 3.1-cm-sized irregular heterogeneous echoic mass within the mammary zone of the right breast (Fig. 2C). There were no enlarged lymph nodes in the axilla. Ultrasound-guided core-needle biopsy was performed to rule out primary breast malignancy or breast involvement of BPDCN. Histologic and IHC features of the breast lesion were similar to those of the right cheek (Fig. 2D); therefore, the lesion was diagnosed as BPDCN of the breast. There was no significant diagnostic challenge.

After two cycles of induction chemotherapy and consolidation chemotherapy (modified hyperCVAD; cyclophosphamide 300-mg/m^2^, vincristine 2-mg/day, daunorubicin 45-mg/m^2^/day, dexamethasone 40-mg/day), the size of the skin and breast lesions markedly decreased and she did not have any adverse effects. After two months, allogenic peripheral blood stem cell transplantation (PBSCT) from a full-matched sibling with conditioning chemotherapy (cyclophosphamide 60-mg/kg/day, busulfan 3.2-mg/kg) was performed. G-CSF and intravenous immunoglobulin (IVIG) therapy were administered after PBSCT. One month after her transplant, she complained of abdominal pain, nausea, and vomiting. The bilirubin and liver enzymes were elevated (total bilirubin, 2.5 mg/dL; AST, 1333 U/L; ALT, 992 U/L) and abdominopelvic computed tomography (CT) scan showed hepatomegaly and periportal edema, which was consistent with acute graft-versus-host disease (GVHD) stage II. There was no evidence of drug toxicity or infection. She was treated by continuing immunosuppression (cyclosporin 150 mg/day) and adding methylprednisolone at 2 mg/kg/day, and she did not experience any major adverse events and continued to do well during and after the hospital stay. She remains in complete remission (CR) without relapse at 34 months since initial diagnosis.

## 3 Discussion

BPDCN is a rare, recently described hematologic malignancy that was initially thought to be derived from natural killer cells (NK-cells) due to its CD56 positivity.^[8]^ After the discovery that BPDCN arises from the precursor cells of plasmacytoid dendritic cells, the current nomenclature (BPDCN) has been settled as a distinct neoplastic entity in the 2008 World Health Organization (WHO) classification of myeloid neoplasms and acute leukemia and remained in the revised 2016 WHO classification.^[9,10]^ The characteristic co-expression of CD4, CD56 and one or more of plasmacytoid dendritic cell-specific markers (CD123 (interleukin-3α-chain), TdT, BDCA2, TCL1, CD138, CD68, Bcl2, etc.) without the expression of T-cell (CD3, CD5), B-Cell (CD19, CD20, CD79a), myeloid (MPO, CD13, CD117), and other NK-cell (CD16, CD57) markers by IHC staining, has been proposed for diagnosing BPDCN.^[11]^

Because BPDCN typically presents with skin lesions, skin biopsy of involved areas is usually helpful for diagnosing BPDCN. However, this case showed rare breast parenchymal involvement of BPDCN without overlying skin lesion. It was incidentally detected by PET-CT with mild FDG-avidity (_max_SUV = 1.9) while previous reports of FDG uptake in cutaneous lesions of BPDCN were in range of 2.4 to 3.5.^[12]^ Therefore, other causes for this weak metabolic lesion besides BPDCN involvement must be evaluated.

Hematologic malignancy affecting the breast is rare because of the paucity of lymphoid tissue in the breast, and the majority are lymphomas. Breast lymphoma typically presents as a solitary oval or round mass with circumscribed to indistinct margin on mammogram.^[13]^ Asymmetries are infrequent (∼20% of cases) and calcifications are almost absent. On ultrasonogram, it is usually depicted as an oval or round mass with nonspecific various margin and hypo- or heterogeneous echogenicity.^[14]^ Our case was demonstrated as nonspecific focal asymmetry without any calcification or skin change on mammogram and presented as an irregular angular heterogeneous echoic mass without significant vascularity or associated lymphadenopathy on ultrasound image. Although no imaging findings of BPDCN of the breast have been reported to date, our case showed slightly different imaging findings with the typical breast hematologic malignancy. Thus, these findings allow it to be categorized as the American College of Radiology (ACR) Breast Imaging Reporting and Data System (BI-RADS) category 4 (suspicious) and it is hard to be distinguished from other malignancy such as invasive ductal carcinoma, ductal carcinoma in situ (DCIS), invasive lobular carcinoma, or sarcoma, or benign diseases of the breast without clinical history taking or core-needle biopsy.

To the best of our knowledge, only 6 cases of breast involvement of BPDCN have been reported to date (Table 1).^[6,7,15–18]^ Since Borchiellini et al^[6]^ reported the first case of BPDCN presented as a breast nodule depicted on CT scan, a few reports have described the cases of BPDCN in the breast region. Chen et al^[7]^ reported a case of a soft tissue mass in the chest wall which was initially misdiagnosed as breast ductal carcinoma but finally confirmed as BPDCN. Kim et al^[15]^ reviewed seven BPDCN cases, and among them, one female patient complained of palpable breast mass at initial presentation which was presented as a subcutaneous mass on CT scan and similar to the case of Borchiellini et al. However, there has been no case demonstrating the radiologic features of BPDCN within the breast parenchyma and this case report might be the first (to our knowledge) to describe the mammogram and ultrasonogram features of BPDCN as a breast mass.

The prognosis is poor because there is no standard treatment for this aggressive disease. Many groups have implemented multiagent chemotherapies such as acute lymphoblastic leukemia/lymphoma regimen (hyperCVAD (course A: cyclophosphamide, vincristine, doxorubicin, dexamethasone, B: methotrexate, cytarabine); VPDL (vincristine, prednisolone, daunorubicin, L-asparaginase); CHOP (cyclophosphamide, doxorubicin, vincristine, prednisone)). Reimer et al^[19]^ reported that high-dose chemo/radiotherapy followed by PBSCT performed in first CR state revealed improved survival, however the median survival rate of patients with BPDCN still appears to be 12–14 months.^[20]^

## 4 Conclusion

In summary, this is the first case demonstrating the rare imaging findings of BPDCN that presented as a breast parenchymal mass, and radiologists and clinicians should be aware of this rare manifestation.

## Acknowledgments

There are no acknowledgments.

## Author contributions

**Conceptualization:** Hyo-jae Lee.

**Data curation:** Hyo-jae Lee, Hye Mi Park.

**Supervision:** Hyo Soon Lim.

**Validation:** So Yeon Ki, Hyo Soon Lim.

**Visualization:** Yoo-Duk Choi, Sook Jung Yun.

**Writing – original draft:** Hyo-jae Lee.

**Writing – review & editing:** Hyo Soon Lim.
